# Fire and brief human occupations in Iberia during MIS 4: Evidence from Abric del Pastor (Alcoy, Spain)

**DOI:** 10.1038/s41598-019-54305-9

**Published:** 2019-12-04

**Authors:** Carolina Mallol, Cristo Hernández, Norbert Mercier, Christophe Falguères, Ángel Carrancho, Dan Cabanes, Paloma Vidal-Matutano, Rory Connolly, Leopoldo Pérez, Alejandro Mayor, Eslem Ben Arous, Bertila Galván

**Affiliations:** 1 UDI de Prehistoria, Arqueología e Historia Antigua, Departamento de Geografía e Historia, Facultad de Humanidades, Universidad de La Laguna, La Laguna, Spain; 2 Archaeological Micromorphology and Biomarker Research Lab, University of La Laguna, La Laguna, Spain; 3 Institute of Archaeomaterials Research, Université Bordeaux Montaigne, Pessac, France; 4 UMR 7194, Département Homme et Environnement, Muséum National d’Histoire Naturelle, Paris, France; 5 Área de Prehistoria, Departamento de Historia, Geografía y Comunicación, Universidad de Burgos, Burgos, Spain; 6 Department of Anthropology, Rutgers University, New Brunswick, USA; 7 Departamento de Ciencias Históricas, Universidad de Las Palmas de Gran Canaria, Las Palmas, Spain; 8 Université Côte-d’Azur, CEPAM, CNRS, Nice, France; 9 Institut Català de Paleoecologia Humana i Evolució Social, Tarragona, Spain; 10 Área de Prehistoria, Universitat Rovira i Virgili, Tarragona, Spain; 11 Departament de Prehistòria, Arqueologia, Història Antiga, Filologia Grega i Filologia Llatina; Facultat de Filosofia i Lletres, Universitat d’Alacant, Sant Vicent del Raspeig, Spain

**Keywords:** Climate-change adaptation, Sedimentology

## Abstract

There is a relatively low amount of Middle Paleolithic sites in Europe dating to MIS 4. Of the few that exist, several of them lack evidence for anthropogenic fire, raising the question of how this period of global cooling may have affected the Neanderthal population. The Iberian Peninsula is a key area to explore this issue, as it has been considered as a glacial refugium during critical periods of the Neanderthal timeline and might therefore yield archaeological contexts in which we can explore possible changes in the behaviour and settlement patterns of Neanderthal groups during MIS 4. Here we report recent data from Abric del Pastor, a small rock shelter in Alcoy (Alicante, Spain) with a stratified deposit containing Middle Palaeolithic remains. We present absolute dates that frame the sequence within MIS 4 and multi-proxy geoarchaeological evidence of *in situ* anthropogenic fire, including microscopic evidence of *in situ* combustion residues and thermally altered sediment. We also present archaeostratigraphic evidence of recurrent, functionally diverse, brief human occupation of the rock shelter. Our results suggest that Neanderthals occupied the Central Mediterranean coast of the Iberian Peninsula during MIS 4, that these Neanderthals were not undergoing climatic stress and they were habitual fire users.

## Introduction

Archaeological evidence across the European continent from the end of MIS 5 through MIS 4 suggests that the cooling effects of the latter in the northern hemisphere influenced the Neanderthal population. There is an apparent reduction in the number of sites suggesting population shrinkage or redistribution^1^. At a regional scale, changes in behaviour have been proposed for Southwestern France, where Neanderthal occupations without fire during MIS 4 have been documented at different sites, motivating a hypothesis on the inability of these Neanderthals to make fire^2–4^. This hypothesis highlights the important role of climate in shaping human behaviour.

To better understand Neanderthal population dynamics and assess the degree of regional behavioural variability during critical conditions of environmental change it is important to seek evidence from the Iberian Peninsula, which has previously been considered as a refugium during cold periods, particularly the south of the peninsula^5–8^ and more recently the east coast as well^9^. Considerable work has been done to establish site-specific palaeoclimatic records for Iberian sites dating to MIS 5^9–13^ and MIS 3, e.g.^14–19^. In contrast, relatively few site-specific records exist for MIS 4 in the peninsula. This potentially hampers our understanding of the local environmental settings in which MIS 4 Neanderthal occupations occurred, as we depend on regional proxy data with coarse resolution.

The Iberian Peninsula is characterised by considerable climatic diversity in which mountainous topography and Atlantic and Mediterranean effects cause local environmental conditions to vary significantly even across relatively short distances^20^. It should be borne in mind, therefore, that local conditions may not always reflect wider regional trends. Marine records (e.g. MD95-2042) suggest that during MIS 4 the Iberian Peninsula saw the progressive development of semi-desert vegetation in line with a significant decrease in sea-surface temperature (SST)^21,22^. Palynological records from Abric Romaní in the northeast indicate millennial-scale climatic variability through the course of MIS 4 into MIS 3, characterised by fluctuations between cold and warm phases driving the decline and expansion of arboreal pollen, including deciduous and evergreen *Quercus*, *Olea* and more^23,24^. This corresponds with terrestrial pollen data from Galicia in the northwest of the peninsula, where records for MIS 4 suggest that coniferous taxa are locally rare at this time, and the presence of *Erica*, *Calluna* and *Poaceae* suggests an environment comprised of alternating heath and temperate grasslands^25^. Meanwhile, evidence from the Tagus Basin in central Iberia interior suggests that episodes of significant loess accumulation during MIS 4 are associated with the development of cold and dry conditions^26^. On the other hand, palynological data from Bolomor Cave in Valencia points to the existence of a glacial refugium during MIS 6, with closed forests and high plant diversity^9^. This evidence could predict similar conditions for MIS 4 in the region and hints at a mosaic scenario for the Iberian Peninsula with different econiches shaped by local geographic factors.

Turning to the archaeological record, more Middle Palaeolithic sites in Iberia are dated to MIS 3 than to MIS 5 and MIS 4 (Supplementary Table S1). As a result, there is considerably more bioanthropological, genetic and cultural information on late Neanderthals (younger than 60 Ka). Although there is an apparent reduction of sites when compared with the preceding and subsequent periods, the significantly shorter duration of the MIS 4 period compared to MIS 5 and MIS 3 and the inaccuracy of some of the chronostratigraphic data should be considered.

The chronostratigraphic and archaeological data from Iberian MIS 4 sites is currently insufficient to understand Neanderthal population dynamics and behaviour in relation to the broader European context or to compare different proxies across MIS 5, MIS 4 and MIS 3 at a regional scale. Regarding fire, there is purported evidence of abundant charcoal, burnt sediment and burnt rocks in Navalmaíllo Layer F (central Spain), but this layer could date to late MIS 5 rather than MIS 4^27^. The lithics from various Iberian MIS 4 sites reflect settlement patterns involving small territories, as informed by short-distance raw material procurement^27–31^ and recurrent occupation of sites^32^.

The central Mediterranean region of Spain is prominent for its Neanderthal archaeological record. There are several sites in the region featuring deep stratigraphic sequences that span either the entire MIS 5 period, including Cova Negra^33^ and Bolomor Cave^34^, or the entire MIS 3 period, such as El Salt^35^. Based on archaeological evidence from these sites, it appears that Neanderthals in this region had similar adaptations during both time intervals. Faunal assemblages are mostly composed of anthropogenic large and medium-sized ungulates, mainly deer, horse and wild goat, with minor proportions of small-sized animals (tortoise and rabbit) and reflect generalist, broad spectrum hunting traditions resulting from adaptation to biodiverse environments. Overall, the lithic technology from these MIS 5 and MIS 3 sites does not show any significant difference either^36–38^. Broadly, it is characterised by a predominance of recurrent centripetal Levallois (RCL) sequences and discoidal schemes, alongside a lesser representation of other Levallois modalities and non-Levallois technical strategies, such as polyhedral and orthogonal procedures^39–42^. This variability, documented throughout MIS 5 and MIS 3 contexts in the region, is possibly related to territorial aspects entailing differential raw material availability and site function determining the use of different activity-specific tools at different sites^43–45^.

Finally, current evidence shows that both MIS 5 and MIS 3 Neanderthals in the central Mediterranean region of Iberia made simple open hearths^35,46,47^ and wood-gathering patterns appear to be similar irrespective of glacial, interglacial, stadial or interstadial conditions^18,48,49^.

Focusing on different aspects of behaviour possibly sensitive to changes in climate such as patterns of group mobility, site occupation duration and seasonality, data from the MIS 5 contexts in this region generally suggest that Neanderthals in the region were occupying relatively small territories, as shown by local lithic raw material procurement patterns^39,50^ and were recurrently occupying sites for short time periods as suggested by tool recycling and diachronic successions of discrete archaeological assemblages in Bolomor Cave^39,51^. In Cova Negra, presence of carnivores throughout the sequence has been interpreted as evidence for short-term human occupations^52^ although no geoarchaeological or archaeostratigraphic data is available to corroborate this. For MIS 3, a lithic raw material study for El Salt shows the continued use of a river-bound territory of less than 5 km throughout the entire sequence^53,54^ and an archaeostratigraphic study of the faunal and lithic record from a segment of the sequence dating to early MIS 3 suggests a pattern of short-term, recurrent occupation of the site^55^. Leierer *et al*.^56^ corroborated this pattern using sedimentary evidence from the fire record and proposed relatively long periods of site abandonment, which could suggest high group mobility. Although these MIS 5 and MIS 3 data are not comparable directly due to their different degrees of temporal resolution, they generally reflect a Neanderthal population consisting of highly mobile groups occupying relatively small territories.

**Figure 1:**
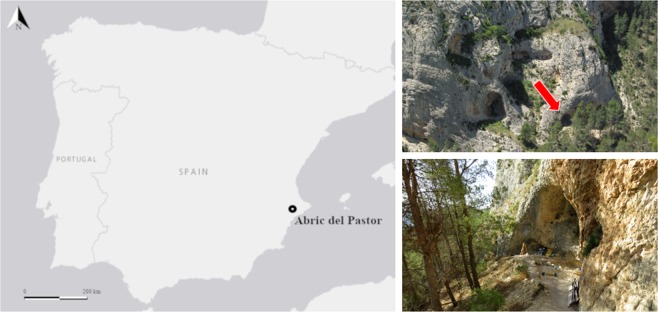
Location of Abric del Pastor rock shelter.

Considering the above-mentioned evidence, filling the gap of information for MIS 4 becomes relevant to: (1) establish the extent of environmental change in the region during MIS 4, and (2) assess the degree of Neanderthal behavioural change (including fire use) from MIS 5 to MIS 3 in a regional context. To this end, here we report archaeological evidence of human occupations with anthropogenic fire and absolute dates from Abric del Pastor, a Middle Palaeolithic rock shelter site in Alcoy, Spain (Fig. 1, Supplementary Fig. S1). The site is undergoing systematic investigation since 2006 and has yielded lithic, faunal and combustion assemblages whose study has provided valuable information regarding Neanderthal settlement dynamics^57,58^. The archaeological sequence appears to represent a succession of brief Middle Palaeolithic occupation events linked to different subsistence activities and these are all centered around single hearths, which are present throughout the sequence^57^. Until now, ascribing the site to a MIS 4 chronological framework has been problematic and the *in situ* nature of the combustion features remained uncertain. A terminus *ante quem* for the sequence is given by the presence of bone remains of *Testudo Hermanni*, which diminishes in the regional record by early MIS 3^59,60^. The good states of preservation of the sedimentary deposit, together with weak frost-induced lenticular microstructures observed microscopically^57^, suggest a generally cool or cold climate but is insufficient evidence to ascribe the sequence to MIS 4. A recent multiproxy study investigating the carbon and hydrogen isotopic values of sedimentary leaf waxes through the sequence (Units I-VI) point to a variable  precipitation regime, while cryoturbation features revealed through soil micromorphology indicate generally cool conditions throughout^56,61^. Based on the anthracological record, the Middle Palaeolithic human occupations are associated with dry/semi-arid supramediterranean conditions (mean annual temperature 8–13 °C and mean annual precipitation 200–600 mm) with juniper stands, heliophilous taxa and scattered cryophilous pine stands^48,49^.

Our ongoing investigations at Abric del Pastor are geared at understanding site formation processes and combustion feature formation processes, dissecting archaeological palimpsests and providing a chronostratigraphic context for the Middle Palaeolithic occupations. In this paper, our objectives are threefold: (1) to provide chronometric dates that help establish if the archaeological deposit formed during MIS 5, MIS 4 or MIS3, and (2) to characterize the combustion features and corroborate their nature as anthropogenic combustion structures, and (3) to describe and assess the variability of the lithic, faunal and combustion record within its stratigraphic context.

## Results

### Absolute dates

**Figure 2:**
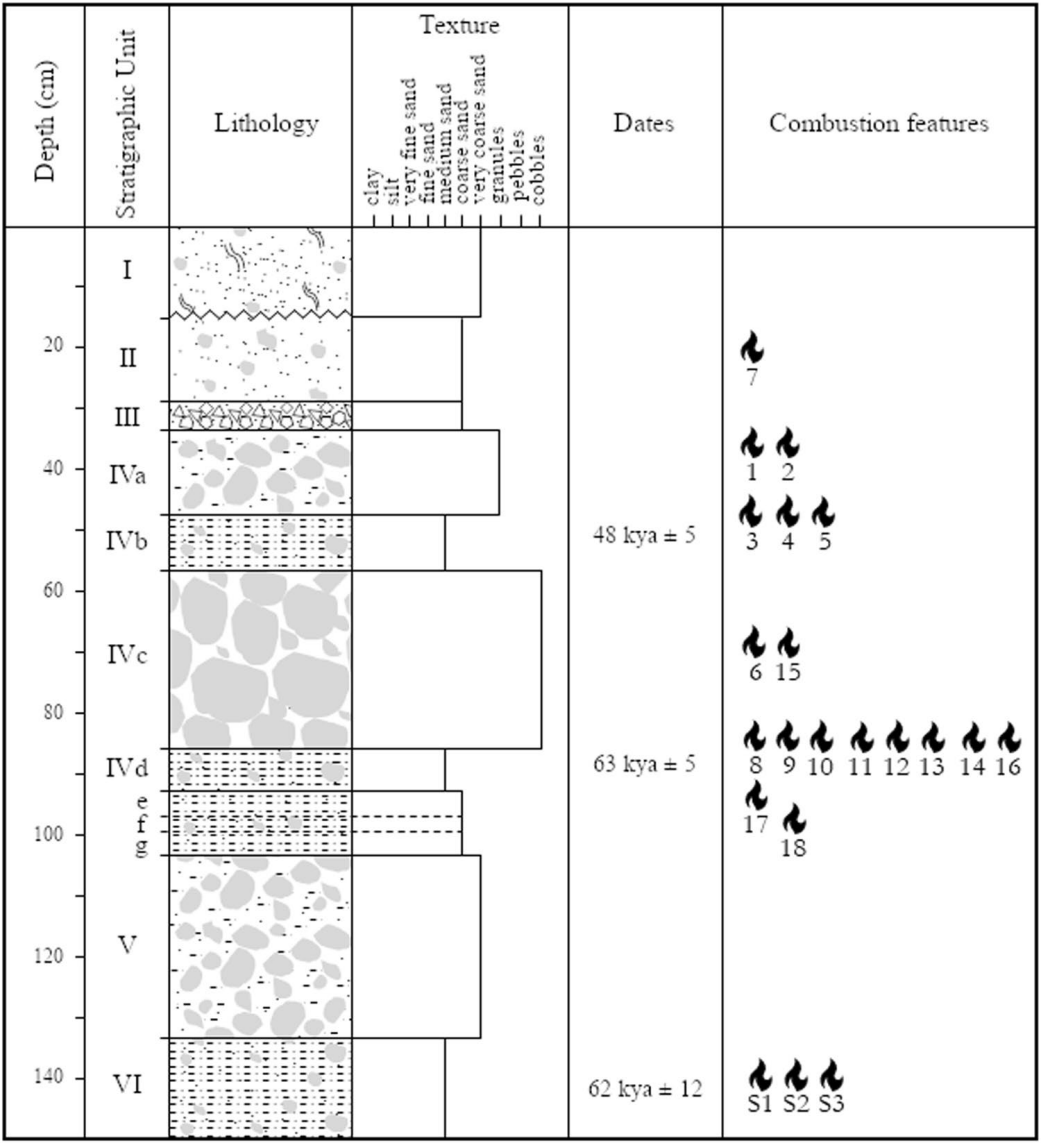
Stratigraphic log of abric del Pastor showing the different lithostratigraphic units, the position of absolute dates and combustion structures.

We report three absolute dates for Stratigraphic Unit IV: (1) an ESR/U-series date (tooth sample) of 48 ± 5 for Stratigraphic Subunit IVb, (2) an OSL date (sediment sample) of 63 ± 5 for Stratigraphic Subunit IVd, and (3) an ESR/U-series date (tooth sample) of 62 + 10/−9 ka for Stratigraphic Subunit VI (Fig. 2; Table 1).

**Table 1:** Absolute dates and associated data for teeth samples AP1601 and AP1602 (ESR/U-Series) and sediment sample OSL1 (OSL) calculated at 1-sigma confidence level.

Sample	Type	Layer	U	Th	K	a-efficiency	Internal	+−	external	+−	annual	+−	De	+−	Age	+−	enamel	±	p-value			
			(ppm)	(ppm)	(%)				dose rate (µGy/a)				(Gy)		(ka)				dentine	±	cement	±
AP1601	Tooth	IV b	1,31	0,70	0,13		326	36	356	23	682	51	33	1	48	5	−0,69	0,12	−0,76	0,12	−49	0,14
OSL1	Sediment	IV d	1,31	0,70	0,13	5	0	0	681	21	681	21	43	1	63	5						
AP1602	Tooth	VI	1,31	0,70	0,13		749	155	378	23	1127	164	70	4,1	62	12/−9	−0,7	0,19	−89	0,15	—	—

### Evidence of anthropogenic fire

**Figure 3:**
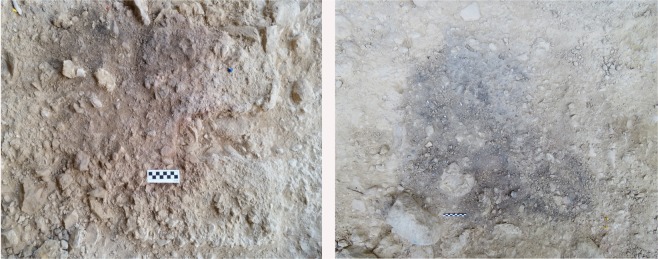
Field views of two Abric del Pastor combustion structures: H2 in Unit IVb (left) and H17 in Unit IVf (right).

Abric del Pastor Stratigraphic Unit IV has yielded numerous burnt bone and flint fragments (Supplementary Table S2) and several sedimentary features with the appearance of combustion structures (Fig. 3). So far, 18 such features have been documented, concentrated in stratigraphic units II (1), IV (15) and VI (2) (see Fig. 2). In the field, these did not exhibit clear ash layers except for H17, which had a 1-2 cm-thick light gray, subcircular layer of silty sediment containing frequent burnt bone fragments. The others were 0.3-1 cm-thick circular or subcircular lenses of dark gray or reddish-brown sediment, with diameters between 0.5 and 1 m.

**Figure 4:**
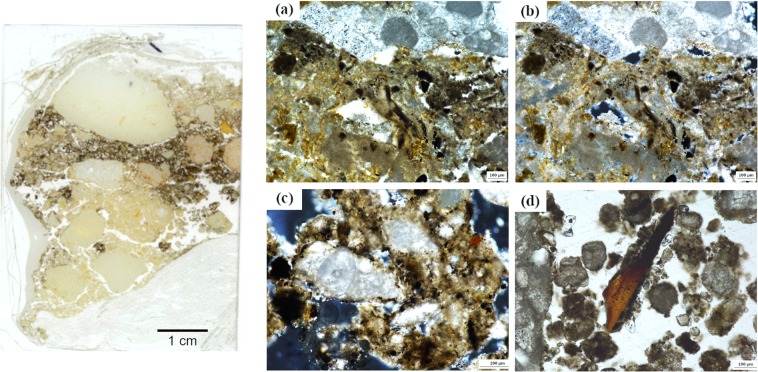
Flatbed scan of a micromorphological thin section from combustion structure H17 in Unit IVf (left image) and selected photomicrographs taken from its black sediment: (**a**) Sedimentary matrix with abundant unidentified charred particles (black) in an ashy matrix (gray). Image taken in plane polarized light; (**b**) Same view in crossed polarized light; c) ashy sediment aggregate with frequent unidentified charred particles. Image taken in plane polarized light; d) Angular burnt bone fragment in plane polarized light.

Micromorphological analysis of several of the combustion features showed the presence of microscopic combustion residues including charcoal, wood ash and burnt bone (Supplementary Table S3 and Fig. S1). The gray layer of H17 shows an aggregated, calcite-rich matrix with frequent unidentified black particles and coatings around clasts, some of which are fissured and rubified (Fig. 4).

The calcitic fraction of the combustion features shows good preservation states. Fourier Transform Infrared Spectroscopy (FTIR) of loose sediment samples from selected combustion features shows calcite as a major mineral component, with minor amounts of clay and quartz (Supplementary Table S4). Highly disordered calcite attributable to wood ash has been identified in several samples using FTIR (Supplementary Fig. S2). The clay content in the site’s sediments is too low to identify clay minerals heated above 500C unambiguously.

**Figure 5:**
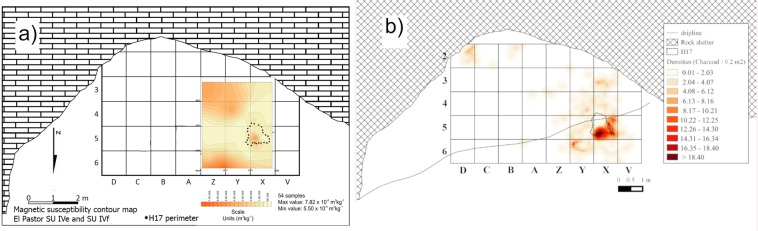
(**a**) Magnetic susceptibility contour map showing a high value zone at the H17 location (the H17 perimeter as recorded in the field is indicated by a dotted line); (**b**) Charcoal distribution contour map showing that most of the charcoal remains in Unit IVf cluster at the H17 location.

Magnetic susceptibility data for the area in and around feature H17 from Stratigraphic Unit IVf indicates burning (Fig. 5a and Supplementary Fig. S3). The values obtained for Unit IVf are low, as expected for a predominantly diamagnetic calcareous sedimentary deposit. However, the center of the feature and some of its adjacent zones yielded high values. The inner part of the combustion feature area and its surroundings is one order of magnitude higher. Anthracological analyses of the Unit IVf charcoal assemblage yielded a well-delimited cluster of strongly fragmented, predominantly coniferous charcoal at the center of the H17 combustion feature. The spatial distribution of this charcoal in conjunction with a zone of high magnetic susceptibility suggests presence of a hearth (Fig. 5b; Supplementary Tables S5 and S6).

**Figure 6:**
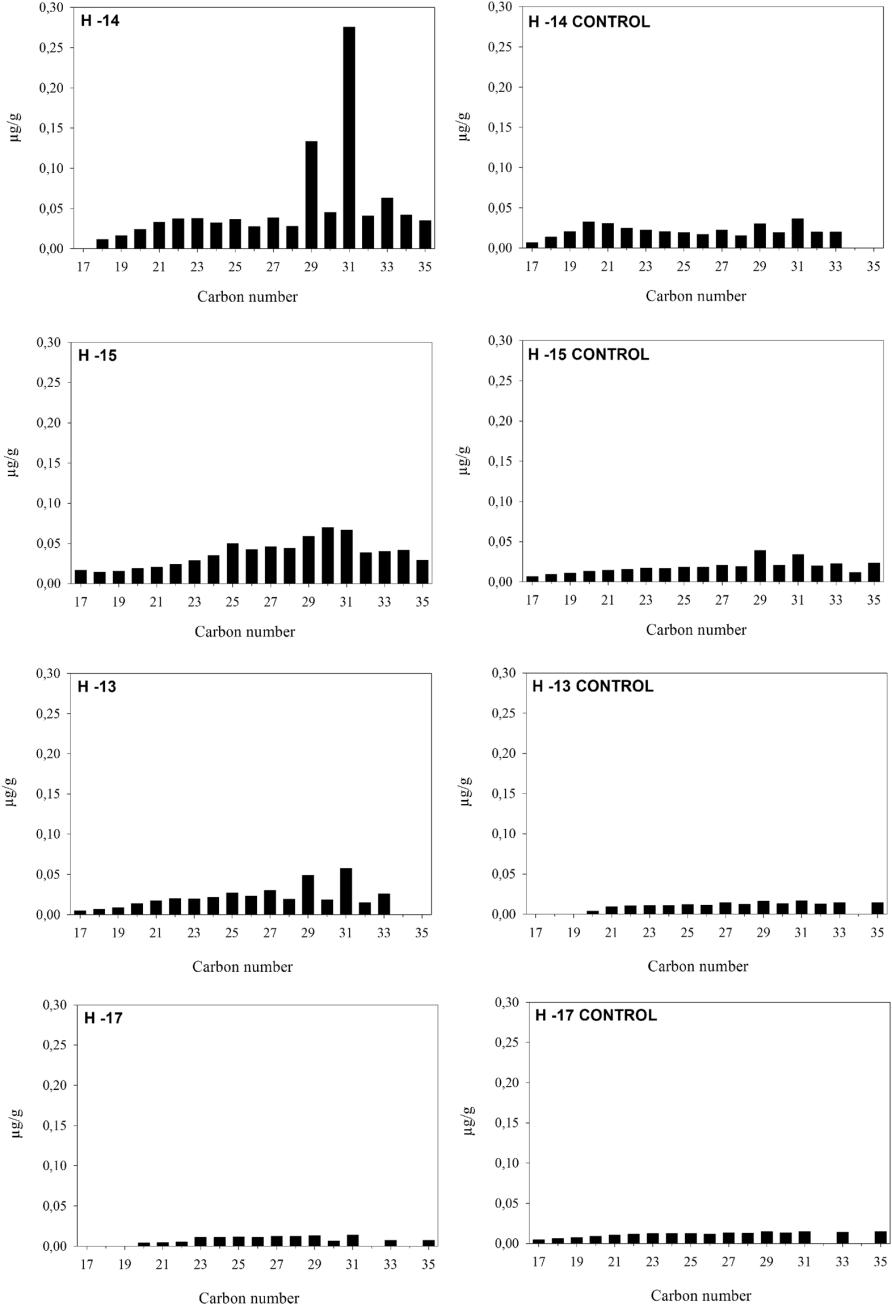
Histograms displaying n-alkane concentrations in combustion features and associated control samples collected from thermally unaltered sediments immediately adjacent (x-axis = number of carbons; y-axis = concentration in μg per g of dry sediment).

Lipid analysis of loose sediment samples collected from selected combustion features yielded *n*-alkane distributions with a predominance of long chain length homologues (nC_27_ – nC_35_), consistent with epicuticular leaf waxes derived from terrestrial higher plants^62^ (Fig. 6, Supplementary Table S7). Several samples yielded distributions with no evident odd-over-even predominance (OEP), which is likely associated with the thermal breakdown of long-chain homologues during combustion^63,64^. Other more polar lipid compounds were identified in the samples (Supplementary Table S8) but are not diagnostic of any specific biological source. It is not possible to establish if these compounds are synchronous with or related to the combustion event, or whether they entered the sediment postdepositionally. Compound specific carbon isotope analyses of long-chain *n*-alkanes nC_29_ and nC_31_ ranged between –34.3‰ to –31.7‰ and –34.3‰ to 32.8‰ respectively. These are values indicative of a vegetation source using the C3 photosynthetic pathway^65^. Interestingly, when compared with control samples collected from outside the combustion features, hearth samples appear offset by ~2‰, which has been recorded elsewhere for *n*-alkane carbon signatures after exposure to high temperatures^64,66^.

### Hearth-related lithic and faunal assemblages from S.U. IVd and IVf

**Figure 7:**
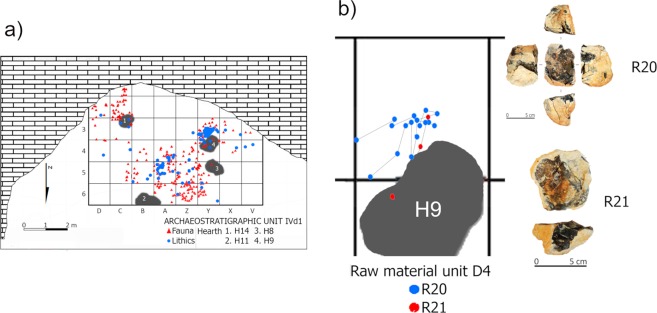
(**a**) Spatial distribution of lithic and faunal remains and combustion structures from Unit IVd1; (**b**) Detailed spatial distribution of the refitted specimens within IVd1 related to combustion structure H9 (left) and a photograph of the refitting sets showing how they conform to an almost whole flint nodule (right).

In the field, the lithic, faunal and combustion remains from Unit IVd appeared to be spatially related (Fig. 7a). Zooarchaeological, technological and spatial analysis of the lithic and faunal record suggests the existence of hearth-related assemblages. The lithic assemblage is dominated by centripetal and preferential Levallois objects, with a minor representation of preferential and unidirectional strategies, discoid, and non-Levallois objects (Supplementary Table S9). Skeletal representation of the IVd faunal record is dominated by cranial remains, mainly scattered bovine and goat teeth. There are also frequent deer long bone fragments and abundant tortoise backplate and plastron plates (Supplementary Table S10). As indicated by technological and zooarchaeological features, different activities were taking place around the hearths. These analyses also show that the hearth-related assemblages were separated in time. First, a lack of bidirectional spatial relationships among different hearth-related lithic objects suggest that they are not synchronous (Fig. 7b). Second, given the small surface area of the rock shelter, the high MNI for Unit IVd (nearly 16 individuals of different taxa) points to several human occupation events.

**Figure 8:**
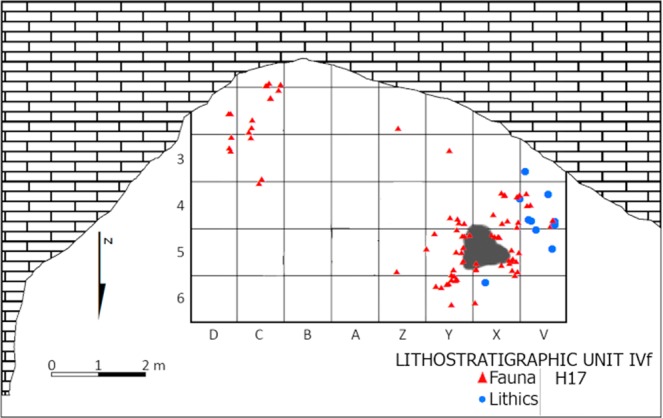
Spatial distribution of lithic and faunal remains associated with H17 within the context of Unit IVf.

Unit IVf is an archaeologically poor deposit comprising a single hearth-related assemblage with frequent, strongly fragmented and variably burnt and unburnt faunal remains (n = 250), and very few (n = 6) small (<0.5 cm) flint flakes (Fig. 8). Except for an accumulation of faunal remains in the Southeast corner of the rock shelter, the bulk of the archaeological remains are concentrated around the H17 combustion feature. The low number of lithic objects and their markedly small size suggests a brief human occupation focusing in tool use rather than knapping activities, which has not been documented in the Iberian or European Middle Palaeolithic record.

## Discussion

Our multidisciplinary study adds a new context to the relatively small set of Middle Palaeolithic sites dated to MIS 4. Considering the error ranges, the absolute dates obtained frame the Abric del Pastor sequence between 43 ka and 72 ka, covering the whole of MIS 4 and early MIS 3. Although the current dates are stratigraphically coherent, more dates need to be obtained to corroborate this chronological framework.

Our proxies of anthropogenic combustion in Stratigraphic Units IV and VI yielded positive results. The sampled sediment contains microscopic combustion residues indicative of burning which, based on their frequency and arrangement were slightly syn- and post-depositionally reworked. The sedimentary structure of Unit IVd is very open and loose and composed almost entirely of limestone gravel, sand and calcitic silt^57^. Thus, individual small-sized particles on this type of substrate can be easily reworked by trampling. In H8 and H9 (Unit IVd), the presence of granular microstructures with sedimentary aggregates containing highly fragmented, silt-sized charcoal and ash and a mix of burnt and unburnt microscopic bone fragments suggest some reworking of the original combustion features. We did not observe any microstratigraphic features indicative of runoff or of any other water-related syn-sedimentary mechanism. Considering this evidence, the prominent absence of ash layers in the Unit IV combustion features - except for H17 in IVf -, could be a result of wind erosion in combination with trampling. This agrees with FTIR data showing random presence of wood ash in the samples, although these data should be taken with caution given the presence of microscopic secondary calcite throughout the Unit IV deposit, as observed in thin section. For now, we have not been able to isolate the signal of this (geogenic) secondary calcite and cannot be certain that it differs from our pyrogenic ash reference samples. Micromorphological samples from combustion features in Unit VI show more abundant combustion residues, including wood ash. These combustion structures, which predate a major roof collapse at the base of Unit IV, are better preserved.

Sediment samples from hearth H17 in Stratigraphic Unit IVf, which appeared in the field as a sub-circular gray zone with a few scattered bone fragments, contain microscopic char. Furthermore, the associated burnt bone remains, charcoal concentrations and magnetic susceptibility data point to *in situ* burning. High magnetic susceptibility values in the adjacent area could be explained by short-distance ash dispersal by wind, trampling or other syn-depositional agents. Charcoal analysis from H17 suggests the use of mainly juniper wood and the concentration of fuel remains at the center of the hearth. Further spatial investigation is needed to assess postdepositional effects on charcoal dispersal.

All the combustion structures presented here (Units IVd, IVf and VI) date to MIS 4. Our integrated analysis of the Unit IVd-f hearth-related assemblages suggests that these represent a succession of brief, functionally diverse human occupation episodes. The Unit IVf assemblage is peculiar in that it could represent a single human occupation without knapping activity. The few lithic objects found (SI-1 Table 1; Fig. 8) are very small in size, predominantly burnt and could be from tool use rather than knapping debris (e.g.^67,68^). Future use-wear analysis could clarify this issue. Our results agree with previous data from stratigraphically higher layers within Stratigraphic Unit IV, particularly Unit IVb, - dated here to early MIS 3-, which was interpreted as a succession of at least three brief human occupation episodes^57^. They also agree with the short-term settlement patterns observed for MIS 3 at El Salt^55,56,69^. El Salt is within 5 km distance from Abric del Pastor and both sites show the same lithic raw material types, all from nearby sources, pointing to exploitation of a single river-bound territory. Together, the current evidence from both sites suggests similar settlement patterns for the Neanderthal groups that occupied the this region of the Iberian Peninsula throughout MIS 4 and 3.

Regarding the palaeoclimatic context, our results also suggest that MIS 4 at Abric del Pastor was not too different from early MIS 3. Recent high-resolution geoarchaeological data point to a variable precipitation regime and generally cool conditions^61^. Previous anthracological data from stratigraphically higher layers within Unit IV (Units IVc)^48^, show a rich diversity of woody taxa, pointing to supramediterranean conditions and readily available woody fuel at the site during the MIS 4 – MIS 3 transition. Instead, MIS 4 Unit IVf shows a reduction in diversity with *Juniperus* sp. as the dominant taxon. However, the unit IVf charcoal sample is associated with a single combustion structure and might reflect anthropogenic selection rather than the natural vegetation. Either way, wood was always an available resource. Further paleoenvironmental evidence from this and other mid latitude MIS 4 Middle Palaeolithic contexts will add to these data and help shape regional paleoenvironmental maps that will allow us to investigate Neanderthal adaptations in more detail.

Here, we have documented the presence of anthropogenic Middle Palaeolithic fire in MIS 4 in the central Mediterranean region of Iberia, which supports the prediction that southern European Neanderthals might have used fire more frequently than in higher latitudes^70^. The associated local climatic context does not appear to have drastically changed during this period and Neanderthal groups living in this region were making fire and show similar settlement patterns to those from the following MIS 3 period. Comparable high-resolution geoarchaeological data is needed for MIS 5 at a regional scale, as well as a geoarchaeological focus on the MIS 5/MIS 4 and MIS4/MIS 3 stratigraphic boundaries at different sites, which might conceal valuable paleoclimatic information and contribute to our understanding of associated human dynamics.

## Methods and Materials

For this study we used two absolute dating techniques: Optically Stimulated Luminescence (OSL) on a loose sediment sample from Stratigraphic Unit IVd and ESR/U-Series on two teeth samples from stratigraphic units IVc and VI (See Supplementary Information for methodological details).

For our multiproxy investigation of the combustion features we used different techniques, which we applied to a selection of features according to their availability for sampling. Most of the features concentrate in Stratigraphic subunit IVd, a rich archaeological horizon. The techniques used were: (1) Soil micromorphology of intact sediment blocks to investigate microscopic components and their arrangement, (2) Fourier Transform Infrared Spectroscopy (FTIR) to identify calcareous wood ash and burnt clay, (3) Magnetic susceptibility to corroborate *in situ* burning, (4) Anthracology to characterize wood fuel and 4) Lipid analysis to seek combustion biomarkers (see Supplementary Information for methodological information on the different techniques).

Integrated analysis of the lithic and faunal record consisted in: (1) Technological analysis following standard analytical parameters, raw material units^71^ and refitting, (2) Zooarchaeological analysis using established standard methods^72,73^, and (3) Spatial analysis of the lithic and faunal record using ArcGIS software (see Supplementary Information for further details).

### Site background and stratigraphic context

Excavations and multidisciplinary investigations at Abric del Pastor have been carried out since 2006 by a research team directed by B.G., C.H. and C.M. The site comprises a small (40 m^2^) rock shelter containing a stratified clastic deposit dominated by coarse components (cobbles and gravel in a sandy matrix).

The Abric del Pastor stratified deposit is 1.3–1.5m-thick and has been subdivided into six lithostratigraphic units, I-VI^57^, of which units II, IV, V and VI have yielded Middle Palaeolithic archaeological remains. The top of the sequence (unit I) is a recent Holocene deposit mainly composed of bioturbated, organic- rich sand with sheep dung. Units II-VI are Pleistocene calcitic deposits hosting the remains of several Middle Palaeolithic human occupation episodes. The bulk of the Middle Palaeolithic evidence retrieved and studied so far comes from Stratigraphic Unit IV, which is in turn comprised of several subunits: IVa-IVg (see Fig. 2). Of these, subunits IVa and IVc are very loose, clast-supported and archaeologically poor, while IVb and IVd are finer-grained and concentrate the bulk of archaeological remains. Excavation of the Unit IV sequence comprised a 38 m^2^ surface area, a large portion of the total space within the small rock shelter. Units V and VI are only known from a 2 × 2 m test pit within the excavation area.

## Supplementary information

Supplementary Information

## Data Availability

The key data generated and/or analysed during this study are included in this published article (and its Supplementary Information files). The complete datasets generated during and/or analysed during the study are available from the corresponding author on reasonable request.
