# Supplementary material for: Fire and brief human occupations in Iberia during MIS 4: Evidence from Abric del Pastor (Alcoy, Spain)
Source: Sci Rep. 2019 Dec 4;9:18281. doi: 10.1038/s41598-019-54305-9 (PMC6892787; doi:10.1038/s41598-019-54305-9)
Supplement: Supplementary file 1 — Supplementary Information [file 41598_2019_54305_MOESM1_ESM.pdf]

Supplementary Information

for

Fire and brief human occupations in Iberia during MIS 4: Evidence from Abric del Pastor (Alcoy, Spain)

Carolina Mallol *[1,2], Cristo Hernández [1], Norbert Mercier [3], Christophe Falguères [4], Ángel Carrancho [5], Dan Cabanes [6], Paloma Vidal-Matutano [7,8], Rory Connolly [1], Leopoldo Pérez [9,10], Alejandro Mayor [11], Eslem Ben Arous [4], Bertila Galván[1]

[1] UDI de Prehistoria, Arqueología e Historia Antigua, Departamento de Geografía e Historia, Universidad de La Laguna, La Laguna, Spain.
[2] Archaeological Micromorphology and Biomarker Research Lab, University of La Laguna, La Laguna, Spain.
[3] Institute of Archaeomaterials Research, Université Bordeaux Montaigne, Pessac, France.
[4] UMR 7194, Département Homme et Environnement, Muséum National d'Histoire Naturelle, Paris, France.
[5] Área de Prehistoria, Departamento de Historia, Geografía y Comunicación, Universidad de Burgos, Burgos, Spain.
[6] Department of Anthropology, Rutgers University, New Brunswick, USA.
[7] Departamento de Ciencias Históricas, Universidad de Las Palmas de Gran Canaria, Las Palmas, Spain.
[8] Université Côte-d'Azur, CEPAM, CNRS, France
[9] Institut Català de Paleoecologia Humana i Evolució Social, Tarragona, Spain.
[10]Àrea de Prehistoria, Universitat Rovira i Virgili (URV), Tarragona, Spain
[11] Departament de Prehistòria, Arqueologia, Història Antiga, Filologia Grega i Filologia Llatina, Universitat d'Alacant, Sant Vicent del Raspeig, Spain

Contents:

1. Extended Data
2. Extended Methods

Extended Data

Figure S1. Photogrammetric 3D model of the Abric del Pastor rockshelter.

Table S1. List of MIS 5, 4 and 3 Middle Palaeolithic Iberian sites. Information obtained from[1] Sala et. al., 2014 unless otherwise cited.

**MIS 3** 67 sites	Abauntz, Abric Romaní, Abrigo del Molino[2], Aitzbitarte III, Amalda[3], Arrillor[4], Axlor, Bajondillo, Bauma dels Pinyons[5], Caldeirão[6], Carihuela[7], Columbeira[8], Cova del Tossal de la Font, Cova del Gegant, Cova del Rinoceront, Cova Foradà, Cova Gran de Santa Linya[9], Cova del Coll Verdaguer[10], Covalejos[11], Cueva Antón[12], Cueva de Los Toros[13], Cueva del Niño[14], Cueva Millán[15], El Castillo, El Mirón, El Pendo, El Salt, El Sidrón, Ermitons, Escoural[6], Esquilleu, Finca de Doña Martina[12], Foz do Exarrique[6], Fuentes de San Cristóbal, Fuente del Trucho, Galería de Las Estatuas[16], Gorham's Cave, Hotel California[2], Higueral de Valleja[17], Hundidero[2], Jarama VI, l'Arbreda, l'Estret de Tragó, La Boja[12], La Güelga, Las Grajas de Archidona, Letzetxiki, la Mina[15], Los Casares[15], Llonín[18], Monforte de Lemos II, Morín, Oliveira[6], Pedreira de Salemas[6], Peña Cabra[15], Peña Miel[19], Prado Vargas, Quebrada[20], Reclau Viver-Mollet, Roca dels Bous, Sima de Las Palomas de Cabezo Gordo, Sopeña, Teixoneres[21], Valdegoba, Vanguard Cave[22], Tossal de La Font, Zafarraya
**MIS 4** 15 sites	Abric Romaní [23], Bajondillo, Benzú, Buena Pinta Cave, Carihuela, Cova del Toll, El Pinar[24], Gorham's Cave, Hotel California [25], Hundidero[26], Las Fuentes[27], Navalmaíllo, Quebrada[28], San Quirce, Vanguard Cave
**MIS 5** 34 sites	Bajondillo[29], Bolomor, Camino Cave, Can Garriga, Carihuela[7], Columbeira[8], Cova 120[30], Cova del Rinoceront, Cova Eirós,Cova Negra, Cueva Antón[12], Cueva Corazón[31], El Castillo, El Pinar[24], Gorham's Cave, Gruta da Oliveira[32], La Callejuela[31], La Ermita[33], L'Estret de Tragó, Lezetxiki[34], Los Aviones[35], Mollet[36], Navalmaíllo, Maltravieso Cave, Nerets, Puig d'Esclats, Quebrada[20], San Luis, San Quirce[31], Tarazona[37], Teixoneres[38], Valdecampaña 4[39], Valdegoba, Vanguard Cave[22]

Table S2. List of faunal and lithic remains from Abric del Pastor Unit IV

Lithostratigraphic Unit	Faunal Remains			Lithic Remains		
	Unburnt	Burnt	Total	Unburnt	Burnt	Total
**II**	5	2	7	2	1	3
**III**	9	0	9	0	0	0
**IVa**	139	4	143	100	5	105
**IVb**	138	5	143	403	3	406
**IVc**	393	22	415	67	4	71
**Ivc-d**	21	3	24	0	0	0
**IVd**	401	70	471	204	10	214
**Ive**	47	0	47	4	1	5
**Ive-f**	64	0	64	1	1	2
**Ivf**	220	30	250	5	1	6
**Ivf-g**	47	4	51	8	4	12
**Ivg**	128	2	130	1	0	1
		TOTAL	1754		TOTAL	825

Table S3. Summary of micromorphological observations from Abric del Pastor combustion features

Sample	Stratigraphic Provenience	Main Features	Interpretation
AP-13-7	IIa-D6/E6, **H7**	Loose, granular microstructure with localized zones of secondary calcite cement (micrite and microspar). Calcitic wood ash aggregates with visible incompletely calcined tissue, charred cells and burnt bone fragments (medium sand sized). 1 bone fragment with partial diagenetic replacement (by unknown mineral).	Reworked combustion
AP-13-9	IVd3/4-Y5/X5, **H8**	Loose, granular microstructure. Poorly preserved charcoal fragments (medium sand-sized), scattered charred cells and charred cell-rich sediment aggregates.	Reworked combustion.
AP-13-8	IVd3/4-Y4/X4, **H9**	Loose, granular microstructure. Poorly preserved charcoal fragments (medium sand-sized), scattered charred cells and charred cell-rich sediment aggregates. Crust: Dense, with vertical fissures and containing in situ broken bone (medium sand-sized) and charred silt-sized cells. Sediment at the lower half of the thin section are largely inorganic.	Reworked combustion. Paleosurface at top of Lev4
AP-13-10	IVd3/4-Z5, **H10**	Loose, spongy/granular microstructure with abundant insect fecal pellets and 1 passage feature (earthworm). 1 cm-sized burnt bone fragment, 1 coprolite fragment, unburnt bone (microfauna), scattered isolated silt-sized charred cells. Fissured gravel.	Bioturbated sediment near combustion structure
AP-13-4	IVd1/2, CONTROL	Loose, granular microstructure. 1 coprolite fragment in Lev1 and 1 burnt bone fragment in Lev2 with char coating.	
AP-13-5	IVd2, CONTROL	Loose, granular microstructure. Rounded wood ash aggregates, isolated charcoal fragments.	
AP-13-11	IVd4, CONTROL	Loose/massive, spongy microstructure. Isolated charred silt-sized cells.	
AP-13-1	VI1/2/3	Lev1-Loose, granular microstructure, gravelly. Fissured gravel. Few rounded calcitic wood ash aggregates, charcoal and charred cells. Lev2-Bedrock gravel. Lev3-Loose, granular microstructure, gravelly. Fissured gravel. Frequent calcitic woodash aggregates and calcitic wood ash cells.	Lev1-Slightly reworked combustion<br>Lev2-In situ combustion

AP-13-2	VI1/2	Lev1-Loose, granular microstructure, gravelly. Abundant rounded calcitic wood ash aggregates (BLUE), 1 leaf ash aggregate (with druses), 1 char fragment, charcoal and charred cells. Lev2- Gravel.	
AP-13-3	VI3, CONTROL	Massive, spongy microstructure, gravelly, largely inorganic.	
AP-15-3	IVd5, C2/C3, **H14**	Loose, crumb/granular microstructure, gravelly, isolated zones of spongy vesicular microstructure, dusty clay coatings and well rounded clasts. Very inorganic.	
AP-15-4	IVd, Z3/Z4, **H13**	Loose, crumb/granular microstructure, gravelly, isolated zones of spongy vesicular microstructure. Cryoturbation features include dusty clay coatings, cappings, platy elongated and occasionally vertically oriented clasts. Very inorganic.	
AP-15-6	IVd5, C2/C3 **H16**	Spongey microstructure, thin dusty clay coatings, well rounded clasts. frequent small burnt bone fragments, occasional fragments of fat derived char	Reworked combustion residues
AP-15-5	IVd5, C2-C3, CONTROL	Loose, crumb/granular microstructure, gravelly, locally spongy vesicular microstructure. Burnt bone fragements, fat derived char and charcoal remains. Cryoturbation features include dusty clay coatings and cappings, rounded and platy clasts. Fresh root matter indicates bioturbation.	Reworked combustion residues
AP-17-MM1	IVf/g, X5/Y5, **H17**	Crumb/granular microstructure, localy massive, gravelly, frequent charred organic matter, burnt bone, charcoal, calcitic wood ash, Fe and Mn segregation, unidentified ‘fatty’ residue	In situ combustion
AP-17-MM2	IVf/g, X5/Y5, **H17**	Crumb/granular microstructure, locally massive, gravelly, frequent charred organic matter, burnt bone, charcoal, calcitic wood ash, Fe and Mn segregation, unidentified ‘fatty’ residue	In situ combustion

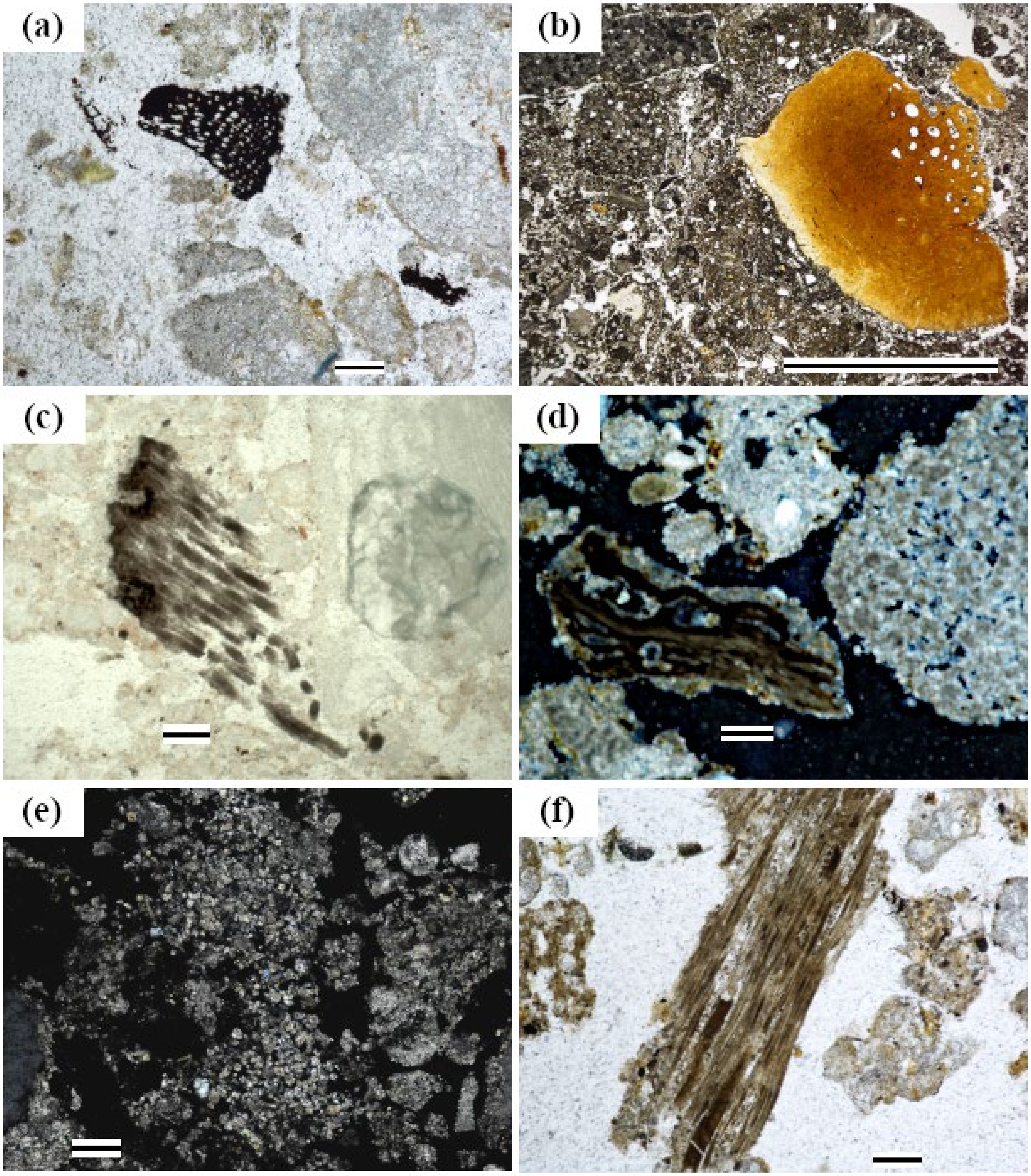

Figure S2. Microscopic combustion residues observed at Abric del Pastor : a) Charcoal, IVd – scale 100 um, PPL [AP-13-9], b) burnt bone, IVd – scale 1 cm, PPL [AP-13-10], c) partially carbonized plant tissue, IVb – scale 100 um, PPL [AP-10-2c], d) charred plant tissue and aggregate of calcareous plant ash, VI – scale 100 um, XPL [AP–13-2], e) calcareous plant ash showing multiple pseudomorphs after calcium oxalate rhombs,

VI – scale 100 um, XPL [AP-10-1], f) partially carbonized plant tissue, VI – scale 100 um, PPL [AP-10-1].

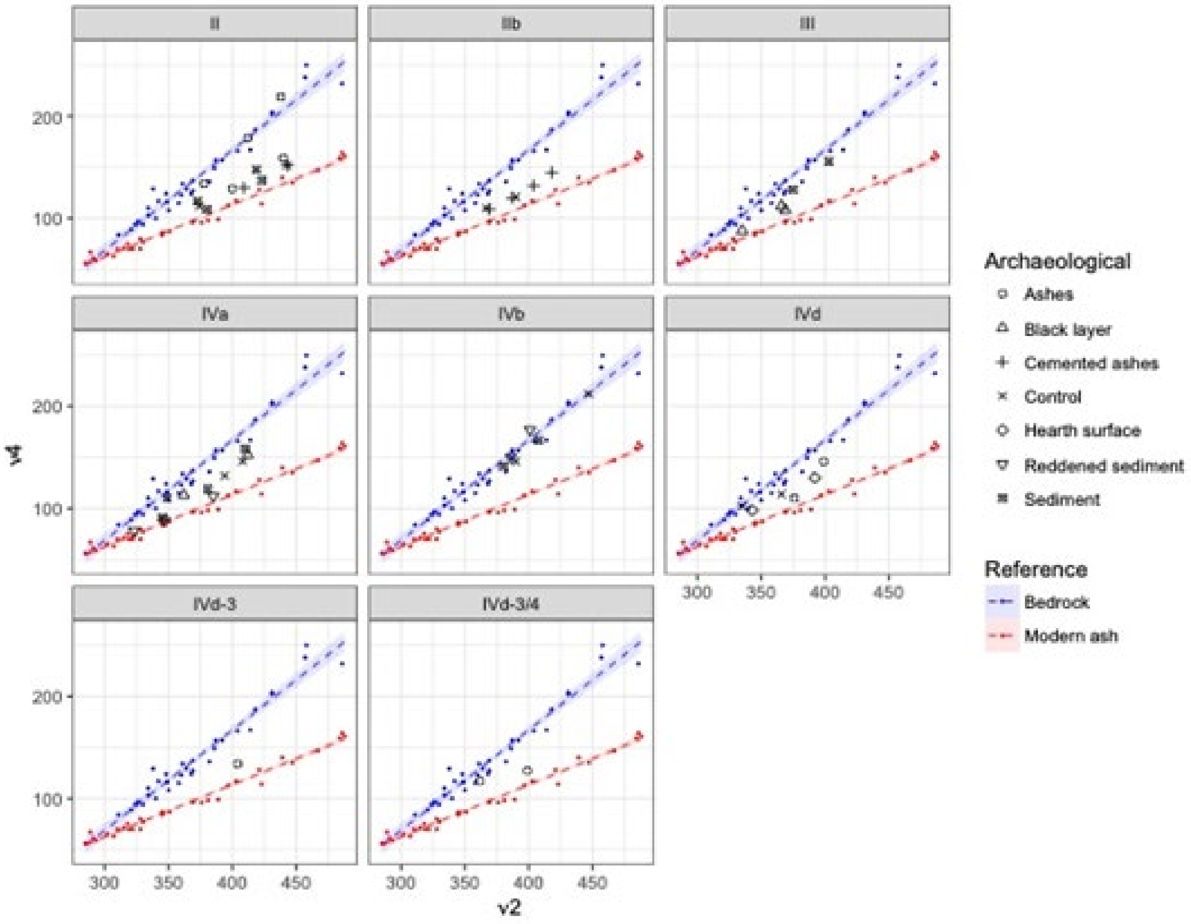

Figure S3. Plot comparing the n2 and n4 normalized peak heights in calcite of the archaeological samples (symbols) vs grinding curves of the reference materials (blue= bedrock; red= modern ash). For the reference samples, the data points show successive grindings, dashed lines indicate the conditional mean, and the shadowed area the model's confidence intervals.

Table S4. FTIR results including interpretation of geogenic/wood ash calcite and unburnt/burnt clay. Legend: Ca: calcite; Cl: clay; Qz: quartz; Dah: dahllite; (b): altered clay – burnt; (nb): unaltered clay – not burnt.

Stratigraphic Unit	Sample	Combustion feature	Field description	FTIR	Calcite
II	AP-13 4	H7	Grayish-yellow silt	Ca, Cl (b?), Qz	Unclear
	AP-13 5	H7	Grayish-yellow silt	Ca, Cl (b?)	Ash
	AP-13 6	H7	Yellow silt adjacent to H7	Ca, Cl (b?), Qz	Ash
	AP-13 7	H7	Yellow silt adjacent to H7	Ca, Cl (b?), Qz	Unclear
	AP-13-2 TP	-	Yellowish-brown sediment	Ca, Cl(b?), Qz	Unclear
	AP-13-5 TP	H7	Yellowish-brown sediment	Ca, Cl(b), Qz	Unclear
	AP-11-4	-	Sediment	Ca, Cl(nb), Qz	Unclear
	AP-11-6	-	Grayish-yellow silt	Ca, Cl(b?)	Unclear
	AP-13-6TP	-	H7 Control sediment	Ca, Cl(b?), Qz	Unclear
	AP-13-7TP	-	H7 Control sediment	Ca, Cl(b?), Qz	Geogenic
IVa	AP-11-1	H1	Reddish-brown sediment	Ca, Cl(nb)	Ash
	AP-11-2	H2	H2 grayish-black sediment	Ca, Cl(b?)	Geogenic
	AP-11-3	H2	H2 grayish-black sediment	Ca, Cl(nb), Qz	Geogenic
	AP-11-5	-	Sediment adjacent to H2	Ca, Cl (b?)	Geogenic
	AP-11-10	-	Sediment adjacent to H2	Ca, Cl(b?), Qz	Ash
IVb	AP-11-7	H3	Reddish-brown sediment	Ca, Cl(nb), Qz	Geogenic

	AP-11-8	H3	Sediment	Ca, Cl(nb), Qz	Geogenic
	AP-11-9	-	Sediment adjacent to H	Ca, Cl(nb), Qz	Geogenic
IVd	AP-13 10	H9	H9 surface (micromorphology sample AP-13-8)	Ca, Cl (b?), Qz	Unclear
	AP-13 11	H9	H9 surface (charcoal-rich) (micromorphology sample AP-13-8)	Ca, Cl (nb), Qz	Geogenic
	AP-13 12	-	Sediment adjacent to H8, H9 and H10	Ca, Cl (b?), Qz	Geogenic
	AP-13 15	-	Sediment adjacent to H8, H9 and H10	Ca, Cl (b?), Qz	Geogenic
	AP-13 1	H11	Grayish-yellow silt near micromorphology sample AP-13-6	Ca, Cl (nb), Qz	Geogenic
	AP-13 2	H11	Grayish-yellow silt near micromorphology sample AP-13-6	Ca, Cl (nb), Qz	Geogenic
	AP-13 3	H11	Grayish-yellow silt near micromorphology sample AP-13-6	Ca, Cl (nb), Qz	Unclear
	AP-13-3 TP	-	Grayish-yellow silt	Ca, Cl(b?), Dah and Qz	Geogenic
	AP-13 13	H9	H9 Grayish-yellow silt (micromorphology sample AP-13-8)	Ca, Cl (nb), Qz	Geogenic
	AP-13 14	H8	Grayish-yellow silt H8 (micromorphology sample AP-13-9)	Ca, Cl (b?), Qz	Geogenic
	AP-13 16	H10	Grayish-yellow silt H10 (micromorphology sample AP-13-10)	Ca, Cl (nb), Qz	Unclear

Table S5. Anthracological data for Unit IVf

**Taxa**	**n**	**%**
Angiosperm	10	4,67
Coniferae	42	19,63
Juniperus sp	127	59,35
cf Juniperus sp	1	0,47
Pinus nigra-sylvestris	23	10,75
Prunus sp.	3	1,40
cf Prunus sp	2	0,93
Quercus sp.	3	1,40
cf Quercus sp.	1	0,47
Quercus sp. evergreen	2	0,93
Total	214	

Table S6. Anthracological data for combustion feature H17 (Unit IVf)

	**White layer**	**Gray Layer**	**Perimeter**	**Total**	
**Taxa**	**n**	**n**	**n**	**n**	**%**
Coniferae	2	7	7	16	14,55
Juniperus sp.	12	31	34	77	70
cf *Juniperus* sp.	1	2		3	2,73
Pinus nigra-sylvestris	1	13		14	12,73
Total	16	53	41	110	100

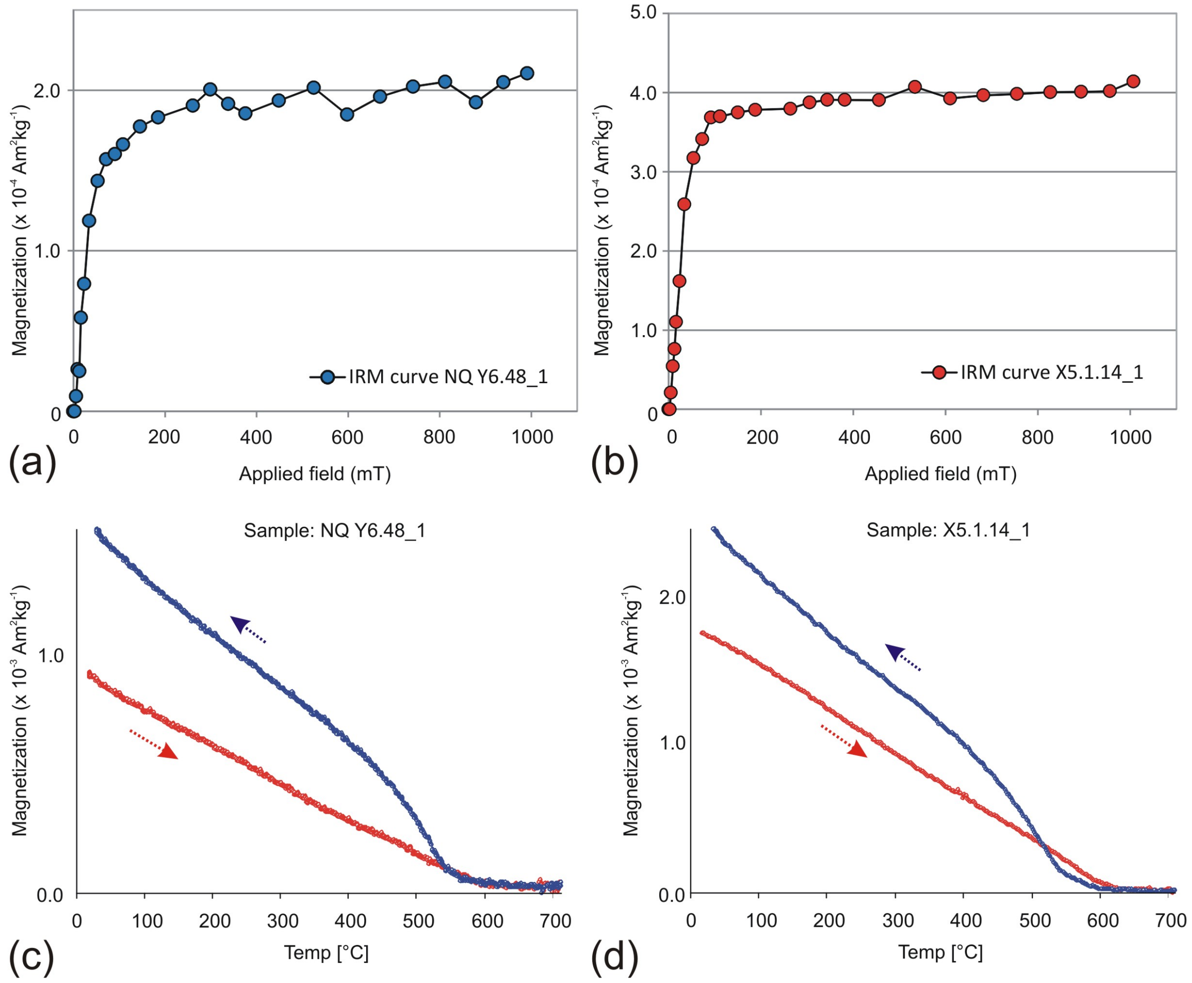

Figure S4. Comparison of magnetic properties between sediment from combustion feature H17 (b, d) and the adjacent sediment (a, c).

Magnetization of sediment adjacent to the combustion feature (Fig. S4 a and c) and sediment from the feature itself (Fig. S4 b and d) is dominated by a low-coercivity mineral (magnetite and/or maghaemite). Their respective IRM acquisition curves are almost saturated between 150 and 300 mT, with a small, high coercivity fraction (5-10%), particularly visible in the unburnt sample, which is not saturated up to 1 T. Thermomagnetic curves (magnetization *vs*. temperature) of both samples show a phase with a Curie temperature of around 580 ºC indicative of magnetite. Both samples formed secondary magnetite upon cooling. The main difference is that the combustion feature sediment (Fig. S4 b and d) exhibits a twofold increase in magnetization intensity in comparison with the adjacent sediment (Fig. S4 b and d).

Table S7. N-alkane data for sediment from Abric del Pastor combustion structures and adjacent (control) sediment

	H13	H13 Control	H14	H14 Control	H15	H15 Control	H17	H17 Control
C_{17}	0.005	0.000	0.000	0.007	0.017	0.007	0.000	0.005
C_{18}	0.007	0.000	0.012	0.014	0.015	0.009	0.000	0.006
C_{19}	0.009	0.000	0.016	0.020	0.015	0.011	0.000	0.008
C_{20}	0.014	0.004	0.024	0.033	0.019	0.013	0.004	0.009
C_{21}	0.018	0.009	0.033	0.031	0.021	0.014	0.005	0.011
C_{22}	0.020	0.010	0.037	0.025	0.024	0.016	0.005	0.012
C_{23}	0.020	0.011	0.038	0.022	0.029	0.017	0.011	0.012
C_{24}	0.022	0.011	0.032	0.020	0.035	0.017	0.011	0.012
C_{25}	0.027	0.012	0.036	0.019	0.050	0.019	0.011	0.013
C_{26}	0.023	0.011	0.028	0.017	0.042	0.018	0.011	0.012
C_{27}	0.030	0.015	0.039	0.022	0.046	0.021	0.012	0.013
C_{28}	0.019	0.012	0.028	0.015	0.044	0.019	0.012	0.013
C_{29}	0.049	0.016	0.133	0.030	0.059	0.039	0.013	0.015
C_{30}	0.018	0.013	0.045	0.019	0.070	0.021	0.006	0.013
C_{31}	0.058	0.017	0.276	0.037	0.067	0.034	0.014	0.015
C_{32}	0.015	0.013	0.041	0.020	0.039	0.020	0.000	0.000
C_{33}	0.026	0.015	0.063	0.020	0.040	0.023	0.007	0.014
C_{34}	0.000	0.000	0.042	0.000	0.042	0.012	0.000	0.000
C_{35}	0.000	0.014	0.035	0.000	0.029	0.023	0.007	0.015
C_{36}	0.000	0.000	0.000	0.000	0.000	0.000	0.000	0.000
C_{37}	0.000	0.000	0.000	0.000	0.000	0.000	0.000	0.000
**Total**	**0.38**	**0.18**	**0.96**	**0.37**	**0.70**	**0.35**	**0.13**	**0.20**

Table S8. Additional lipid compounds identified in sediment samples from Abric del Pastor combustion structures and adjacent (control) sediment

Sample	Alcohols	Fatty Acids	Other Compounds
H13	1-Octadecanol, TMS derivative	Hexadecanoic acid, methyl ester	Oleanitrile
		9, 12-Octadecadienoic acid, methyl ester	Stigmastan-3,5-diene
		Methyl stearate	9-Octadecenamide, (z) (Oleamide)
		Tridecanedioic acid, dimethyl ester	
H13C		Octadecanoic acid, 9, 10, 12-trimethoxy, methyl ester	Oleanitrile
		Hydroxydecapentadecanoic acid, methyl ester	
H14	1-Tetradecanol, TMS derivative	Palmitic acid, TMS derivative	Stigmasta-3,5-diene
	1-Pentadecanol, TMS derivative		
	1-Hexadecanol, TMS derivative		
	1-Octadecanol, TMS derivative		
H14C		13-Docosenoic acid, methyl ester	
H15		Hexadecanoic acid, methyl ester	
		Methyl stearate	
H15C	1-Hexadecanol, TMS derivative	Palmitic acid, TMS derivative	Benzamide, N-(2-cyanocylopent-1-enyl)-3,4 dimethoxy
		Stearic acid	
		Petroselenic acid, TMS derivative	
		n-Hexadecanoic acid	
		Octadecanoic acid	
H17		Octadecanoic acid	9-Octadecenamide, (z) (Oleamide)
		Hexadecanoic acid, 1-(hydroxymethyl)-1,2-ethandiyl ester	
		Hexadecanoic acid, methyl ester	
H17C		Methyl stearate	
		Hexadecanoic acid, methyl ester	
		Oleic acid	

Table S9. Technological data for the Unit IVd lithic assemblage, including raw material units, refitting sets and reduction sequence phases identified. Abbreviations: RMU=raw material unit; CR=cortex removal; PREP=core preparation; BP=blank production; DP=débitage products; FRC=final reduction of the core

Raw Material (flint) Type	RMU	Total	Refitting Sets	Refitted Objects	Reduction Sequence Phases				
					CR	PREP	BP	DP	FRC
Beniaia	D1	4	1	2	-	-	3	-	1
Serreta	D2	2	1	2	1	-	1	-	-
Serreta	D3	11	1	2	-	4	3	2	-
Beniaia	D4	77	2	21	32	9	28	6	2
Serreta	D5	36	3	11	12	11	-	9	-
Serreta	D6	3	1	3	2	-	-	1	-
Beniaia	D7	2	-	-	-	-	1	1	-
Serreta	D8	3	-	-	3	-	-	-	-
Serreta	D9	3	-	-	-	1	1	1	-
Mariola	D10	1	-	-	-	1	-	-	-
Mariola	D11	3	-	-	2	1	-	-	-
Serreta	D12	2	-	-	-	-	2	-	-
Burnt (indeterminate)	Indeterminate	3	-	-	1	1	-	1	-
Beniaia (1.85%) Mariola (29.63%) Serreta (59.26%) Indeterminate (9.26%)	Indeterminate	54	-	-	6	3	15	11	2

Table S10. Zooarchaeological data for Unit IVd. Abbreviations : NR= Number of remains ; NISP=Number of Identified Remains ; MNE=Minimal Number of Elements ; MNIf= minimal Number of Individuals by Frequency ; MNIc=Minimal Number of Individuals by Combination

Order	NR	NISP	MNE	MNI f	MNI c
Artiodactyla	73	73	59	7	10
Avifauna	2	2	2	1	1
Carnivora	1	1	1	1	1
Lagomorpha	8	8	8	2	4
Perissodactyla	1	1	1	1	1
Testudines	129	129	46	4	4
Large Size	2	-	1	-	-
Medium Size	80	-	5	-	-
Small Size	32	-	3	-	-
Very Small Size	5	-	1	-	-
Undetermined	136	-	3	-	-
Total	469	214	130	16	21

Extended Methods

Micromorphology

16 intact sediment blocks were collected from different possible combustion features and control sediment adjacent to them between 2010 and 2016. Plaster of Paris was used to extract them without any physical disturbance. 9 cm x 6 cm thin sections were manufactured by Thomas Beckmann (Schwülper-Lagesbüttel, Germany). The thin sections were observed using a Nikon Eclipse E200 polarizing microscope with magnifications ranging between 20 and 100 and described following the standard guidelines of Stoops[40] and Nicosia and Stoops[41].

Fourier Transform InfraRed Spectroscopy (FTIR)

29 loose sediment samples were collected from the combustion features and adjacent sediments (control) in 2011 and two different field seasons within 2013. The samples were analyzed using an iS5 FT-IR Nicolet Thermo Scientific Spectrometer. Infrared spectra were obtained using KBr pellet at 4 cm^{-1} resolution. Clays exposed to high temperatures were identified following Berna, et al.[42]. This method was only used when the volume of clay minerals in the samples was sufficient to reflect changes in the specific absorption bands of the clay spectrum. The origin of calcite, i.e. geogenic or anthropogenic (i.e. ashes), was determined following the grinding curves method[43,44]. Note that secondary calcite has been identified in thin section. We

believe that the microcrystalline structure of this calcite could limit our ability to establish the origin of calcite using its atomic order.

Magnetic susceptibility

54 bulk sediment samples (~ 15 g) were collected from in and around feature H17 (stratigraphic unit IVf) in order to study magnetic susceptibility variation. Between 4 and 7 samples per square ($1m^2$) were collected covering an area of around 9 m^2. Each sample was sieved at 2 mm, 1 mm and 500 μ to separate clasts from the sedimentary fraction. Subsequently, cylindrical (3.6 cm^3) plastic containers were filled and weighed to measure the low-field magnetic susceptibility at room temperature in a KLY-4 susceptometer (AGICO, noise level 3 x 10^{-8} S.I.). Since not all the samples were completely filled the MS results are shown on a mass-specific basis. Additionally, two different samples (~ 450 mg) were selected to study magnetic properties in detail. One was collected from feature H17 (Sample X5.1.14_1, square X5; MS = 5.53 x 10^{-8} m^3kg^{-1}) and the other from the surrounding area (sample NQ.Y6.48_1, square Y6, MS = 8.49 x 10^{-9} m^3kg^{-1}). The analyses were carried out with a Variable Field translation balance and included the measurement of progressive isothermal remanent magnetization (IRM) acquisition curves, hysteresis loops (± 1T), backfield coercivity curves and thermomagnetic curves up to 700 ºC in air. *Rock_Mag_Analyzer* software[45] was used to interpret these data. All the analyses were performed at the laboratory of Palaeomagnetism of Burgos University, Spain.

Lipid Analysis

Sediment samples were first dried at 60 °C for 48 hours and subsequently homogenised using an agate pestle and mortar which had previously been washed with MeOH and dichloromethane (DCM). All non-volumetric glassware used for lipid extraction were thoroughly cleaned, solvent washed with MeOH and calcined at 450 °C for 10 hours prior to extraction. Lipids from 5g sediment samples were extracted in 40mL dichloromethane/methanol (DCM:MeOH 9:1) by ultrasonic extraction (three cycles of 30 minutes) and centrifugation (three cycles at 4700 rpm). The total lipid extract (TLE) was then concentrated under a steady flow of N_2 gas at 40 °C in a rotary evaporator. The TLE was subsequently reconstituted using DCM and separated into fractions of differing polarity by solid phase extraction (SPE) through a silica gel column (1g silica, 70-230 mesh and 0.1g sand 50-70 mesh, both previously calcined at 450 °C for 10 hours).

All fractions were analysed by gas chromatography with a coupled detection and mass-selective detector (GC-Agilent 7890B, MSD Agilent 5977A) equipped with a HP-5MS capillary column (30m, ID: 250 μm, film thickness 0.25 μm). A temperature program with an initial temperature of 70 °C for 2 min and a heating rate of 12 °C/min to 140 °C, and a final temperature of 320 °C with a heating rate of 3 °C/min for 3 min, using a Helium carrier gas (2 ml/min) was employed. The PVT injector was held at a split

ratio of 5:1 at an initial temperature of 70 °C for 0.85 min and heated to 300 °C at a programmed rate of 720 °C/min.

Dosimetry on site

A series of 10 Al_2O_3:C dosimeters were inserted in the different stratigraphic units (from I to VI) and remained in place for five months. Their doses were measured following the procedure described by Kreutzer et al.[46]. The deduced dose rates (gamma + cosmic) do not vary significantly throughout the stratigraphy and are quite low, in agreement with the calcareous nature of the sediments (from 317 to 388 µGy/a). These external dose rates were used for calculating the ages of the tooth and sediment samples.

Dated Sediment sample

A sediment sample was collected from layer IVd in absolute darkness and dated using Optically Stimulated Luminescence (OSL) dating[47]. Quartz grains in the 60-80µm fraction were extracted by sieving and purified with acids (HCl and H_2SiF_6). The Single Aliquot Regenerative (SAR) dose protocol[48] was applied to large aliquots (6mm in diameter) for measuring the fast component of the OSL signal. First, a preheating plateau test was performed, indicating no dependence of the equivalent doses (De) on temperature (between 200 and 260°C). Subsequently, a dose recovery

test yielded a value close to one (1.02 ±0.02). Finally, a series of 20 aliquots were analyzed and a representative De was calculated using the Central Age Model[49].

In the laboratory, gamma spectrometric analysis allowed determination of U,Th, K contents in the sediment, from which the alpha and beta external dose rates received by the 60-80µm grains were calculated.

Dated Teeth

Two herbivorous teeth were selected for combined ESR/ U-series dating[50]: an Equidae premolar from Stratigraphic Unit IVc (AP1601) and a *Bovinae* tooth from the Stratigraphic Unit VI (AP1602). Enamel, dentin and cement present in AP1601 were separated mechanically and the radio-isotope content in the dentin and cement was measured by alpha-ray spectrometry at the Institut de Paléontologie Humaine (IPH), Paris, using standard methods[51], and by gamma-ray spectrometry[52].

ESR measurements were performed on a EMX Bruker ESR spectrometer at the Institut de Paléontologie Humaine (IPH), Paris using the following parameters: 1mW microwave power, 0.1mT modulation amplitude, room temperature (19°C), 10mT scan range, 4min scan time and 100kHz frequency modulation. At least three measurements were repeated for each dose on different days. The equivalent doses (D_E) were determined from the asymmetric enamel T1-B2 signal at g = 2.0018[53] and fitted from the experimental data using an exponential function (SSE) with Microcal Origin Pro 8 software with $1/I^2$ weighting. The D_E were calculated from mean values obtained from

3 repeated measurements for each aliquot. ESR age calculations were carried out using the ESR-DATA program of Grün[54] which uses an alpha efficiency of 0.13 ± 0.02 (Grün and Katzenberger-Apel, 1994) and Monte-Carlo beta attenuation factors[55] based on the thickness of the tooth enamel and outer layers removed.

Lithic Analysis

Technological analysis was carried out from a techno-economic perspective. Raw material type classification was based on parameters from previous macroscopic and microscopic analyses[56]. Subsequently, flint objects within each raw material class were grouped into different raw material units (RMU)[57] representing specific reduction sequences on single flint blanks. Finally, refitting analysis was performed, first within each RMU and subsequently across RMUs to check for possible errors in RMU classification. The final step was morpho-technological characterization of all the artefacts.

Spatial Analysis

Spatial analyses were performed using geographic information systems (GIS) software (ArcGIS ® ArcMap v10.2.2 and ArcScene v10.2.2) to plot georeferenced lithic and faunal remains and combustion features. Taphonomic and field information, such as the different strata's topography were taken into consideration for subsequent spatial analysis. Archaeologically sterile strata and combustion features were used as

archaeostratigraphic landmarks to locate former surfaces and potential human occupation surfaces, respectively. Raw material units and refitting sets were also considered and played a critical role in our archaeostratigraphic analysis [58]. They were three-dimensionally plotted and analysed through longitudinal profiles and cross-sections across the entire excavation area. Note that at Abric del Pastor, the excavated area comprises almost the entire sheltered zone, including an area presently outside rockshelter's dripline but possibly sheltered in the Pleistocene.

References

1. Sala, R., i Roura, E. C., de Castro, J. M. B. & Arsuaga, J. L. *Pleistocene and Holocene hunter-gatherers in Iberia: the current archaeological record*. (Universidad de Burgos y Fundación Atapuerca, 2014).
2. Álvarez-Alonso, D., de Andrés-Herrero, M., Díez-Herrero, A., Medialdea, A. & Rojo-Hernández, J. Neanderthal settlement in central Iberia: Geo-archaeological research in the Abrigo del Molino site, MIS 3 (Segovia, Iberian Peninsula). *Quat. Int.* **474**, 85–97 (2018).
3. Marín-Arroyo, A. B. *et al.* Chronological reassessment of the Middle to Upper Paleolithic transition and Early Upper Paleolithic cultures in Cantabrian Spain. *PLoS One* **13**, e0194708 (2018).
4. Iriarte-Chiapusso, M. J., Wood, R. & de Buruaga, A. S. Arrillor cave (Basque Country, northern Iberian Penisula). Chronological, palaeo-environmental and cultural notes on a long Mousterian sequence. *Quaternary International* **508**, 107–115 (2019).
5. Vaquero M, A. E. *Memòria arqueològica de l'excavació d'urgència de Bauma dels Pinyons (Capellades, Anoia).* (Centre d'Informació i Documentació del Patrimoni Cultural,

Barcelona, 2010).
6. Pereira, T., Haws, J., Bicho, N. O Paleolítico Médio no território portugués. *Mainake* **XXXIII**, 11–30 (2011).
7. Carrión, J. S. *et al.* The sequence at Carihuela Cave and its potential for research into Neanderthal ecology and the Mousterian in southern Spain. *Quaternary Science Reviews* (2019). doi:10.1016/j.quascirev.2019.04.012
8. Zilhão, J., Cardoso, J. L., Pike, W. G. & Weninger, B. Gruta Nova da Columbeira (Bombarral, Portugal): site stratigraphy, age os the Mousterian sequence, and implications for the timing of Neanderthal extinction in Iberia. (2011).
9. Mora, R. *et al.* Contextual, technological and chronometric data from Cova Gran: Their contribution to discussion of the Middle-to-Upper Paleolithic transition in northeastern Iberia. *Quat. Int.* **474**, 30–43 (2018).
10. Daura, J. *et al.* Palaeoenvironments of the last Neanderthals in SW Europe (MIS 3): Cova del Coll Verdaguer (Barcelona, NE of Iberian Peninsula). *Quat. Sci. Rev.* **177**, 34–56 (2017).
11. Jones, J. R., Richards, M. P., Reade, H., de Quirós, F. B. & Marín-Arroyo, A. B. Multi-Isotope investigations of ungulate bones and teeth from El Castillo and Covalejos caves (Cantabria, Spain): Implications for paleoenvironment reconstructions across the Middle-Upper Palaeolithic transition. *Journal of Archaeological Science: Reports* **23**, 1029–1042 (2019).
12. Zilhão, J. *et al.* Precise dating of the Middle-to-Upper Paleolithic transition in Murcia (Spain) supports late Neandertal persistence in Iberia. *Heliyon* **3**, e00435 (2017).
13. Montes, L., Utrilla, P., Martínez-Bea, M. Trabajos recientes en yacimientos musterienses de Aragón: una revisión de la transición Paleolítico medio/superior en el Valle del Ebro. *Zona Arqueológica* **7**, 215–232. (2006).
14. García-Moreno, A. *et al.* Revisión y estudio multidisciplinar del yacimiento de la Cueva del

Niño (A\`yna, Albacete). in *Actas de la primera reunión científica de arqueología de Albacete* 253–270 (Instituto de Estudios Albacetenses‘ Don Manuel’. Excma. Diputación de Albacete, 2016).

15. Alcaraz-Castaño, M. *et al.* A context for the last Neandertals of interior Iberia: Los Casares cave revisited. *PLoS One* **12**, e0180823 (2017).
16. Arsuaga, J. L. *et al.* Evidence of paleoecological changes and Mousterian occupations at the Galería de las Estatuas site, Sierra de Atapuerca, northern Iberian plateau, Spain. *Quat. Res.* **88**, 345–367 (2017).
17. Jennings, R. P. *et al.* New dates and palaeoenvironmental evidence for the Middle to Upper Palaeolithic occupation of Higueral de Valleja Cave, southern Spain. *Quat. Sci. Rev.* **28**, 830–839 (2009).
18. Sanchis, A. *et al.* Neanderthal and carnivore activities at Llonin Cave, Asturias, northern Iberian Peninsula: Faunal study of Mousterian levels (MIS 3). *C. R. Palevol* **18**, 113–141 (2019).
19. Montes, L., Utrilla, P. & Hedges, R. Le passage Paléolithique Moyen-Paléolithique Supérieur dans la vallée de l’Ebre (Espagne). Datations radiométriques des grottes de Peña Miel et Gabasa. *Trabalhos de Arqueologia* **17**, 87–102 (2001).
20. Carrión-Marco, Y. C. *et al.* Climate, environment and human behaviour in the Middle Palaeolithic of Abrigo de la Quebrada (Valencia, Spain): The evidence from charred plant and micromammal remains. *Quat. Sci. Rev.* (2018).
21. Talamo, S. *et al.* The Radiocarbon Approach to Neanderthals in a Carnivore Den Site: a Well-Defined Chronology for Teixoneres Cave (Moià, Barcelona, Spain). *Radiocarbon* **58**, 247–265 (2016).
22. Doerschner, N. *et al.* Chronology of the Late Pleistocene archaeological sequence at Vanguard Cave, Gibraltar: Insights from quartz single and multiple grain luminescence dating. *Quaternary International* **501**, 289–302 (2019).

23. Burjachs, F. *et al.* Palaeoecology of Neanderthals during Dansgaard–Oeschger cycles in northeastern Iberia (Abric Romaní): From regional to global scale. *Quat. Int.* **247**, 26–37 (2012).
24. Casabó J Rovira M. El Pinar, yacimiento al aire libre con industria sobre lascas del Paleolítico Medio. Avance preliminar. in *Aragón-litoral Mediterráneo: intercambios culturales durante la Prehistoria. Homenaje a Juan Maluquer de Motes.* (ed. Utrilla, P.) 89–95 (Institución Fernando el Católico, Zaragoza, 1992).
25. Arnold, L. J., Demuro, M., Navazo, M., Benito-Calvo, A. & Pérez-González, A. OSL dating of the Middle Palaeolithic Hotel California site, Sierra de Atapuerca, north-central Spain. *Boreas* **42**, 285–305 (2013).
26. Navazo, M. *et al.* Hundidero: mis 4 open air neanderthal occupations in Sierra de Atapuerca. *Archaeology, Ethnology and Anthropology of Eurasia* **39**, 29–41 (2011).
27. Aparicio, J. Primeras dataciones de C-14 para el musteriense valenciano. *Archivo de Prehistoria Levantina* **16**, 9–38 (1981).
28. Villaverde, V. *et al.* Diachronic variation in the Middle Paleolithic settlement of Abrigo de la Quebrada (Chelva, Spain). *Quaternary International* **435**, 164–179 (2017).
29. Cortés Sánchez, M. Cueva Bajondillo (Torremolinos). *Secuencia cronocultural y paleoambiental del Cuaternario reciente en la Bahıa de Málaga. Málaga: CEDMA* 546 (2007).
30. Agustí, B. *et al.* La cova 120, parada de caçadors-recol\textperiodcentered lectors del paleolític mitjà. *Cypsela: revista de prehistòria i protohistòria* 7–20 (1991).
31. Domingo, R., Peña-Monné, J. L., de Torres, T., Ortiz, J. E. & Utrilla, P. Neanderthal highlanders: Las Callejuelas (Monteagudo del Castillo, Teruel, Spain), a high-altitude site occupied during MIS 5. *Quat. Int.* **435**, 129–143 (2017).
32. Deschamps, M. & Zilhão, J. Assessing site formation and assemblage integrity through stone tool refitting at Gruta da Oliveira (Almonda karst system, Torres Novas, Portugal): A

Middle Paleolithic case study. *PLoS One* **13**, e0192423 (2018).
33. Sánchez Yustos, P. & Diez Martín, F. Dancing to the rhythms of the Pleistocene? Early Middle Paleolithic population dynamics in NW Iberia (Duero Basin and Cantabrian Region). *Quat. Sci. Rev.* **121**, 75–88 (2015).
34. Falgueres, C., Yokoyama, Y., Arrizabalaga, A. La geocronología del yacimiento Pleistocénico de Lezetxiki (Arrasate, País Vasco). Críticas de las dataciones existentes y algunas nuevas aportaciones. *Munibe* **57**, 93–106 (2005).
35. Hoffmann, D. L. *et al.* U-Th dating of carbonate crusts reveals Neandertal origin of Iberian cave art. *Science* **359**, 912–915 (2018).
36. Maroto, J., Julià, R., López-García, J. M. & Blain, H.-A. Chronological and environmental context of the Middle Pleistocene human tooth from Mollet Cave (Serinyà, NE Iberian Peninsula). *Journal of Human Evolution* **62**, 655–663 (2012).
37. Caro-Gómez, J. A., Del Olmo, F. D., Artigas, R. C., Espejo, J. M. R. & Barrera, C. B. Geoarchaeological alluvial terrace system in Tarazona: Chronostratigraphical transition of Mode 2 to Mode 3 during the middle-upper pleistocene in the Guadalquivir River valley (Seville, Spain). *Quat. Int.* **243**, 143–160 (2011).
38. Tissoux, H. *et al.* Datation par les séries de l'Uranium des occupations moustériennes de la grotte de Teixoneres (Moia, Province de Barcelone, Espagne). *Quaternaire* 27–33 (2006). doi:10.4000/quaternaire.613
39. Martín, F. D., Yustos, P. S. & González, J. Á. G. La ocupación paleolítica en los páramos del Duero. Nuevos datos procedentes de Valdecampaña (Olivares de Duero, Valladolid). *Zephyrvs* **62**, (2009).
40. Stoops, G. Guidelines for analysis and description of soil and regolith thin sections. *Sort* **100**, 250 (2003).
41. Nicosia, C. & Stoops, G. *Archaeological Soil and Sediment Micromorphology*. (John Wiley & Sons, 2017).

42. Berna, F. & Goldberg, P. Assessing Paleolithic pyrotechnology and associated hominin behavior in Israel. *Isr. J. Earth Sci.* **56**, 107–121 (2007).

43. Poduska, K. M. *et al.* Decoupling local disorder and optical effects in infrared spectra: differentiating between calcites with different origins. *Adv. Mater.* **23**, 550–554 (2011).

44. Regev, L., Poduska, K. M., Addadi, L., Weiner, S. & Boaretto, E. Distinguishing between calcites formed by different mechanisms using infrared spectrometry: archaeological applications. *J. Archaeol. Sci.* **37**, 3022–3029 (2010).

45. Leonhardt, R. Analyzing rock magnetic measurements: The RockMagAnalyzer 1.0 software. *Comput. Geosci.* **32**, 1420–1431 (2006).

46. Kreutzer, S. *et al.* Environmental dose rate determination using a passive dosimeter: Techniques and workflow for α-Al2O3: C chips. *Geochronometria* **45**, 56–67 (2018).

47. Aitken, M. J. *Introduction to Optical Dating: The Dating of Quaternary Sediments by the Use of Photon-stimulated Luminescence*. (Clarendon Press, 1998).

48. Wintle, A. G. & Murray, A. S. Quartz OSL: Effects of thermal treatment and their relevance to laboratory dating procedures. *Radiat. Meas.* **32**, 387–400 (2000).

49. Galbraith, R. F., Roberts, R. G., Laslett, G. M., Yoshida, H. & Olley, J. M. Optical dating of single and multiple grains of quartz from Jinmium Rock Shelter, Northern Australia: Part I, Experimental design and statistical models. *Archaeometry* **41**, 339–364 (1999).

50. Grün, R. & Katzenberger-Apel, O. An alpha irradiator for ESR dating. *Ancient TL* **12**, 35–38 (1994).

51. Bischoff, J. L., Rosenbauer, R. J., Tavoso, A. & de Lumley, H. A test of uranium-series dating of fossil tooth enamel: results from Tournal Cave, France. *Appl. Geochem.* **3**, 145–151 (1988).

52. Yokoyama, Y. & Nguyen, H. V. Direct and non destructive dating of marine sediments, manganese nodules and corals by high resolution gamma-ray spectrometry. in *Isotope marine chemistry* 259–289 (Uchida Rokakuho Tokyo, 1980).

53. Grün, R., Joannes-Boyau, R. & Stringer, C. Two types of CO_2^- radicals threaten the fundamentals of ESR dating of tooth enamel. *Quat. Geochronol.* **3**, 150–172 (2008).
54. Grün, R. The DATA program for the calculation of ESR age estimates on tooth enamel. *Quat. Geochronol.* **4**, 231–232 (2009).
55. Brennan, B. J., Rink, W. J., McGuirl, E. L., Schwarcz, H. P. & Prestwich, W. V. Beta doses in tooth enamel by 'one-group' theory and the ROSY ESR dating software. *Radiat. Meas.* **27**, 307–314 (1997).
56. Molina, F. J. Estudio geoarqueológico de entornos sedimentarios fluvio-lacustres y endorreicos con industrias del Paleolítico medio en el norte de la provincia de Alicante (España). *Recerques del Museu d'Alcoi* **25**, 7–29 (2016).
57. Conard, N. J. & Adler, D. S. Lithic Reduction and Hominid Behavior in the Middle Paleolithic of the Rhineland. *Journal of Anthropological Research* **53**, 147–175 (1997).
58. Machado J, Mayor A, Hernández CM, Galván B. Lithic refitting and the analysis of Middle Palaeolithic settlement dynamics: a high-temporal resolution example from El Pastor rock shelter (Eastern Iberia). *Archaeol. Anthropol. Sci.* **accepted**, (2019).
